# Integrative proteomic analysis reveals the potential diagnostic marker and drug target for the Type‐2 diabetes mellitus

**DOI:** 10.1007/s40200-025-01562-3

**Published:** 2025-01-22

**Authors:** Zhen Jia, Ning Jiang, Lin Lin, Bing Li, Xuewei Liang

**Affiliations:** 1 Department of Peripheral Vascular Diseases, First Affiliated Hospital, Heilongjiang University of Traditional Chinese Medicine, Harbin, China; 2 Department of Cardiovascular Medicine, First Affiliated Hospital, Heilongjiang University of Traditional Chinese Medicine, Harbin, China; 3 Department of Radiology, First Affiliated Hospital, Heilongjiang University of Traditional Chinese Medicine, Harbin, China

**Keywords:** Diabetes mellitus, T2DM, Proteome profiles, Predictive markers

## Abstract

**Objective:**

The escalating prevalence of Type-2 diabetes mellitus (T2DM) poses a significant global health challenge. Utilizing integrative proteomic analysis, this study aimed to identify a panel of potential protein markers for T2DM, enhancing diagnostic accuracy and paving the way for personalized treatment strategies.

**Methods:**

Proteome profiles from two independent cohorts were integrated: cohort 1 composed of 10 T2DM patients and 10 healthy controls (HC), and cohort 2 comprising 87 T2DM patients and 60 healthy controls. Differential expression analysis, functional enrichment analysis, receiver operating characteristic (ROC) analysis, and classification error matrix analysis were employed.

**Results:**

Comparative proteomic analysis identified the differential expressed proteins (DEPs) and changes in biological pathways associated with T2DM. Further combined analysis refined a group of protein panel (including CA1, S100A6, and DDT), which were significantly increased in T2DM in both two cohorts. ROC analysis revealed the area under curve (AUC) values of 0.94 for CA1, 0.87 for S100A6, and 0.97 for DDT; the combined model achieved an AUC reaching 1. Classification error matrix analysis demonstrated the combined model could reach an accuracy of 1 and 0.875 in the 60% training set and 40% testing set.

**Conclusions:**

This study incorporates different cohorts of T2DM, and refines the potential markers for T2DM with high accuracy, offering more reliable markers for clinical translation.

**Supplementary Information:**

The online version contains supplementary material available at 10.1007/s40200-025-01562-3.

## Introduction

Diabetes mellitus (DM), a complex chronic systemic disease involving metabolic disorders, is categorized into two subtypes, type-1 DM (T1DM) and type-2 DM (T2DM), with T2DM accounting for about 95% of individuals [1]. T2DM arises from a complex interplay of factors, notably insulin resistance (IR) and deficiency in insulin secretion, and is characterized by dysregulation of carbohydrate, lipid and protein metabolism [2]. In addition, as reported, T2DM is associated with various complications affecting multiple organs, such as diabetic cardiac autonomic neuropathy, diabetic retinopathy, osteoporosis, and acute complications [3].

Although rapid development in DM research over the last few decades [4–7], several clinical problems remain to be addressed, especially in biomarker discovery. For instance, currently, available biomarkers do not provide a reliable and stable predictive power for the early diagnosis or the identification of seemingly healthy individuals at a higher risk of disease development in the future. Moreover, current research needs additional adjuvant strategies to improve the clinical decision-making in patients with T2DM, rather than relying solely on glucose measurements. Most importantly, using glucose readings alone does not fit well with personalized medicine, where using an algorithm of several variables is more powerful than one [8].

Ultra-sensitive and high-throughput mass spectrometry (MS)-based proteomic measurement technology has enabled large-scale proteomic analysis of diverse types of clinical samples, especially for blood samples [9–12]. Peripheral blood sampling could be readily evaluable for biomarker identification, due to the easiest implementation in routine clinical practice. Proteins circulating in the blood also used to reflect the whole-body states, which were used for biomarker applications [13–15]. Although many proteins were reported to be associated with T2DM [16–18], there is still a lack of the integrative analysis to reveal the reliability of these proteins.

Due to the heterogeneity in clinical samples, we integrated the proteomic datasets from the two cohorts related to T2DM. This approach enabled us to encompass a broader spectrum of clinical variability within our analyses. By conducting integrative proteomic analysis, we aimed to identify a reliable marker to effectively distinguish T2DM patients from healthy controls, bolstering result robustness and generalizability.

## Methods

### Data source and processing

The Mass Spectrometry (MS)-based proteome expression profiles used in this study includes the proteome data of the circulating extracellular vesicle (EV) from a cohort composed of people with normal glucose tolerance (NGT, also referred as HC, *N* = 10), and T2DM patients (*N* = 10) (named as cohort 1) [19], and the serum proteome data from a cohort composed of HC (*N* = 60) and T2DM patients (*N* = 87) (named as cohort 2) [20]. The T2DM group in cohort 1 comprised 5 males and 5 females (average age: 50.7 years), and the HC group had 5 males and 5 females (average age: 45.9 years). In the cohort 2, the T2DM group consisted of 33 males 54 females with an average age of 52.1 years, and the HC group had 23 males and 37 females with an average age of 51.1.

### Differential protein and pathway analysis

Differential expression analysis between HC and T2DM groups was conducted using the two-sided Wilcoxon rank-sum test. The significantly differentially expressed proteins were defined as differential proteins with at least twofold change (FDR < 0.05) (Supplementary Table 1 and 2); Pathway enrichment analysis was performed by ConsensusPathDB (CPDB, http://cpdb.molgen.mpg.de) [21, 22] based on the differentially expressed proteins in each group. Pathways with *P*-value less than 0.05 were regarded to be significant enrichment (Supplementary Table 1 and 2).

### Protein–protein interaction (PPI) network

A PPI network was constructed to elucidate protein correlations using the Search Tool for the Retrieval of Interacting Genes (STRING; http://string-db.org) (version 11.5) online database [23]. The PPI network was visualized using Cytoscape software [24].

### Identifying potential markers for T2DM diagnosis

To explore the potential markers for predicting T2DM diagnosis, we screened the commonly candidates significantly differentially expressed in healthy controls and T2DM groups (fold changes > 2 or < 0.5; FDR < 0.05) in the two cohorts for the further analysis. The ROC analysis (pROC R package version 1.16.2 and Caret R package version 6.0–86) was employed to assess the predictive effect of the potential markers based on the reported methods [25]. In addition, samples of cohort 1 were randomly partitioned into 60% of samples (used as the 60% training set) and the remaining 40% (representing the 40% testing set) for model evaluation (including sensitivity, specificity, and accuracy) using the tenfold cross validation [25].

### Exploration of the potential markers with exercise times

We incorporated the proteome profiles from T2DM with different exercise times, including baseline, AM, PM training [26]. We performed differential analysis among the three groups (baseline, AM, and PM) using ANOVA test.

### The association of the potential markers across diverse tissues and cancers

We explored the protein expression of the potential markers across diverse tissues, and the protein and RNA expression of the potential markers across diverse cancer types, based on the Human Protein Atlas (HPA, https://www.proteinatlas.org/).

### Statistics and reproducibility

﻿Standard statistical tests including but not limited to Wilcoxon rank-sum test and ANOVA test were used for proteome data analysis. Statistical significance was set at *P* < 0.05. All analyses were conducted using R (v3.5.1) and GraphPad Prism 8 software, with validation by two statisticians. The Wilcoxon rank-sum test was used to examine the differentially expressed proteins between healthy controls and T2DM groups. The ANOVA test was used to examine whether proteins were differentially expressed among baseline, AM, and PM groups.

## Results

### Study design and baseline clinical characteristics

**Fig. 1:**
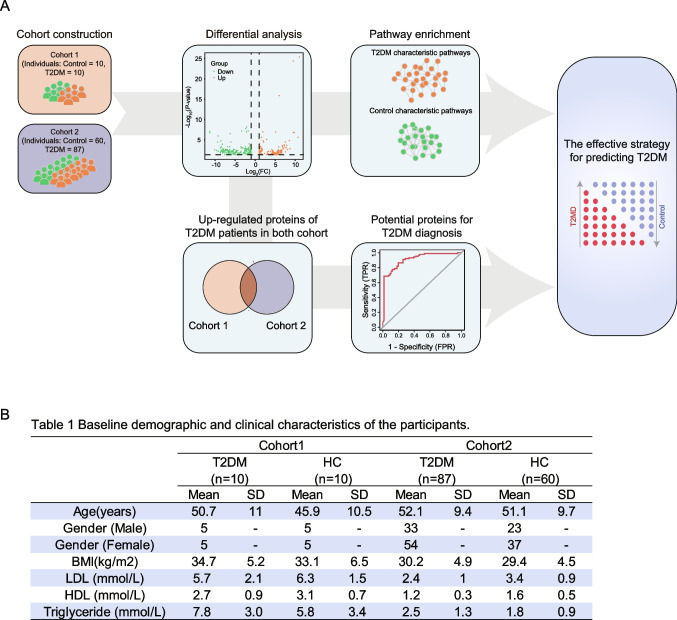
Summary of the plasma proteomic analysis of CRC cohort and healthy control. **A** Overview of the data analysis workflow. **B** Table 1: Baseline demographic and clinical characteristics of the participants

To detect reliable peripheral markers for T2DM, we incorporated two independent cohorts and performed the combined analysis based on the two proteomic datasets (Fig. 1A). For the cohort 1, 20 participants (10 T2DM patients and 10 HC) were included in this study [20]; for the cohort 2, 147 participants were included, which was composed of 87 T2DM and 60 HC [19]. The detailed baseline information of the cohort 1 and cohort 2 have been previously described. The baseline clinical characteristics of T2DM and HC groups included in this study were summarized in Fig. 1B (Table 1). In the two cohorts, we found there was no significant difference in age, gender, and body mass index (BMI) between T2DM and HC groups.

### Proteomic features associated with T2DM

**Fig. 2:**
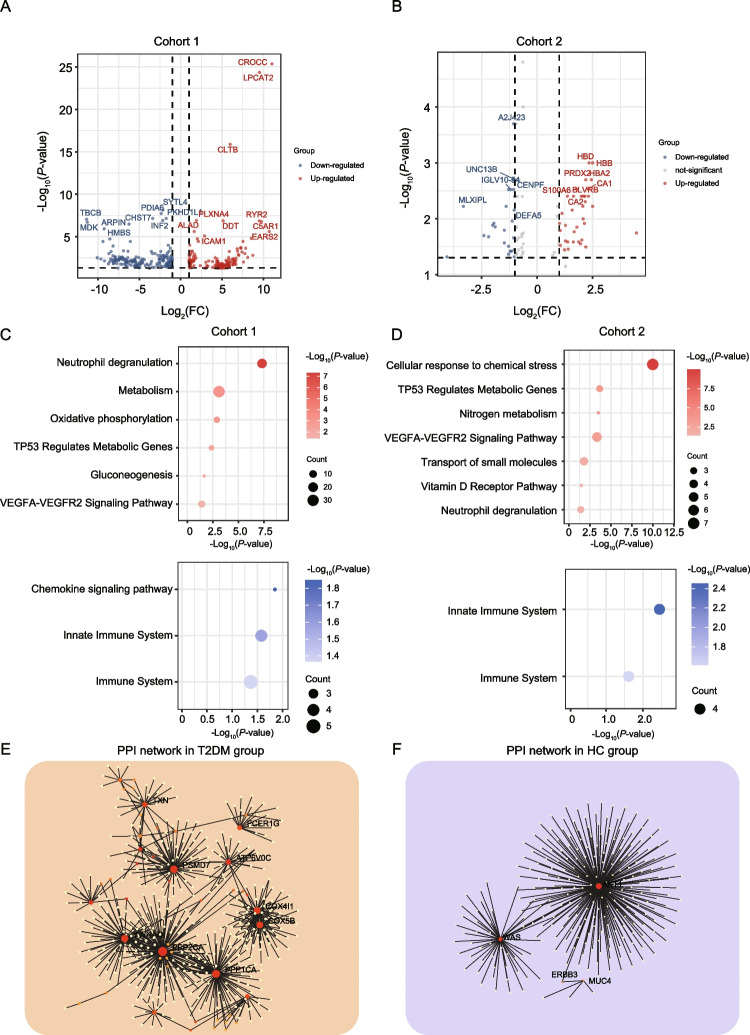
The differential proteome analysis between T2DM and HC groups. **A**-**B** Volcano showing the differential expressed proteins (DEPs) in the cohort 1 (A) and cohort 2 (B). **C**-**D** Bubble plot showing the CPDB pathway enrichment in the T2DM (upper) and HC (down) groups, in the cohort 1 (C) and cohort 2 (D). **E**–**F** PPI networks of hub proteins up-regulated in the T2DM (E) and HC (F) groups

To gain insight into the biological alteration associated with T2DM and to identify potential biomarkers for T2DM, we applied a strict differential analysis strategy with at least twofold change and FDR < 0.05 to characterize the extracellular vesicle (EV) proteome data in the cohort 1 and the serum proteome data in the cohort 2. In the cohort 1, comparative proteomic analysis identified 308 differentially expressed proteins (DEPs). Among these DEPs, 144 DEPs were significantly increased in the T2DM group, and 164 DEPS were significantly decreased in the T2DM group (Fig. 2A). Furthermore, we determined the dominant bioprocesses associated with T2DM based on these DEPs through ConsensusPathDB (CPDB) molecular interaction data obtained from 31 different public repositories. As a result, we found 144 DEPs up-regulated in T2DM group, were mainly involved in neutrophil degranulation, VEGFA-VEGFR2 signaling pathway, and metabolic bioprocesses (oxidative phosphorylation, TP53 regulates metabolic genes, and gluconeogenesis (*P* < 0.05); while 164 DEPs down-regulated in T2DM group, were enriched in immune related pathways, including chemokine signaling pathway, and innate immune system (Fig. 2C).

In the cohort 2, we also applied same analysis strategy, and identified 61 DEPs between the T2DM group and HC group (Fig. 2B). Of these DEPs, 36 DEPs showed a significant increase in the T2DM group, which were mainly enriched in neutrophil degranulation, cellular response to chemical stress, VEGFA-VEGFR2 signaling pathway, transport of small molecules, vitamin D receptor pathway, and metabolic bioprocesses (TP53 regulates metabolic genes and nitrogen metabolism); while 25 DEPs showed a significant decrease in the T2DM group, which were mainly enriched in innate immune system and immune system (Fig. 2D). Furthermore, combined the changed proteins and pathways in the two cohorts, we constructed and visualized PPI networks based on the STRING online database [23, 27, 28], which has been adopted in many biological researches, especially in proteomic analysis for the interaction network construction and hub protein identification [29–31]. The PPI network identified hub proteins, of which the hub proteins, including TXN, PSMD7, FCER1G, ATP6V0C, COX4I1, COX5B, YWHAQ, PPP2CA, and PPP1CA, were dominant in the T2DM group, highlighting the importance function of these hub proteins in the biological regulation of the T2DM development (Fig. 2E), while AKT1 and WAS were dominant in the HC group (Fig. 2F). Therefore, the PPI network not only interpreted the complex biological signaling more effectively, but also revealed the clusters and hubs within the network.

### Predictive model for T2DM diagnosis

**Fig. 3:**
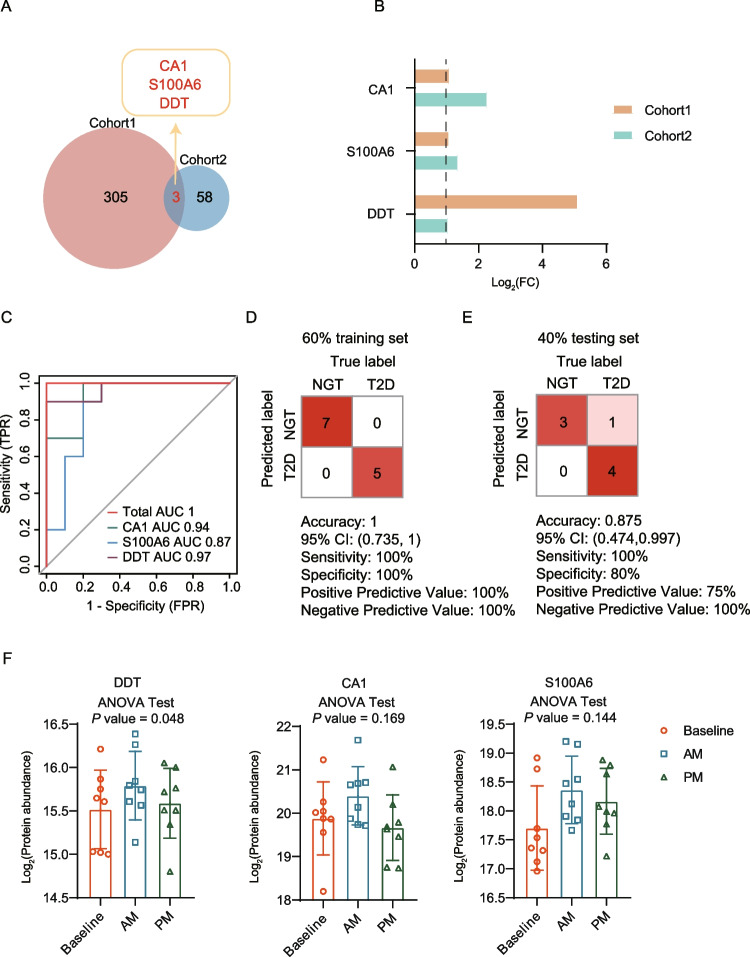
The predictive model for T2DM patients. **A** Venn showing the overlapped DEPs in the cohort 1 and cohort 2. **B** The differential expression of CA1, S100A6, and DDT in the cohort 1 and cohort 2. **C** ROC curves of the single protein, and the panel of the three proteins in predicting T2DM diagnosis. **D**-**E** Classification error matrix showing the accuracy, sensitivity, and specificity of the predictive model in training set (D) and testing set (E). **F** The differential expression of CA1, S100A6, and DDT among the three groups (baseline, AM, and PM)

Based on the DEPs identified in the cohort 1 and cohort 2, we firstly refined a group of proteins, including CA1, S100A6, and DDT, showed significant up-regulation in the both two cohorts (Fig. 3A-B). Then, we evaluated the predictive effect of the three proteins for T2DM diagnosis. We found the three proteins had a good prediction with AUC values of 0.94 (CA1), 0.87 (S100A6), 0.97 (DDT). Further combined predictive effect of the predictive model composed of the three proteins could reach the best performance with an AUC of 1 **(**Fig. 3C**)**, To train and subsequently test the model, samples were partitioned on the basis of T2DM and HC groups (60% training set and 40% testing set). We applied cross-validation to the training set to evaluate the predictive results. The accuracy metrics showed that the predictive model, comprising the three proteins CA1, S100A6, and DDT, exhibited high prediction, with accuracy of 1, sensitivity of 100% and specificity of 100% in the training set, and with accuracy of 0.875, sensitivity of 100% and specificity of 80% in the testing set **(**Fig. 3D-E**)**. As reported, exercise training is commonly prescribed interventions for individuals with type 2 diabetes to combat metabolic disease and improve glucose homeostasis [32]. Then, we incorporated the proteome profiles from a cohort of T2DM patients with different exercise times, including baseline, AM, PM training [26]. We performed differential analysis among the three groups (baseline, AM, and PM), and evaluated the association of the three proteins with exercise training. As a result, we found the High-Intensity Interval Training (HIT) in AM could lead higher levels of the three proteins (DDT, CA1, and S100A6) than in PM, indicating a greater impact of AM versus PM training on the proteome change.

**Fig. 4:**
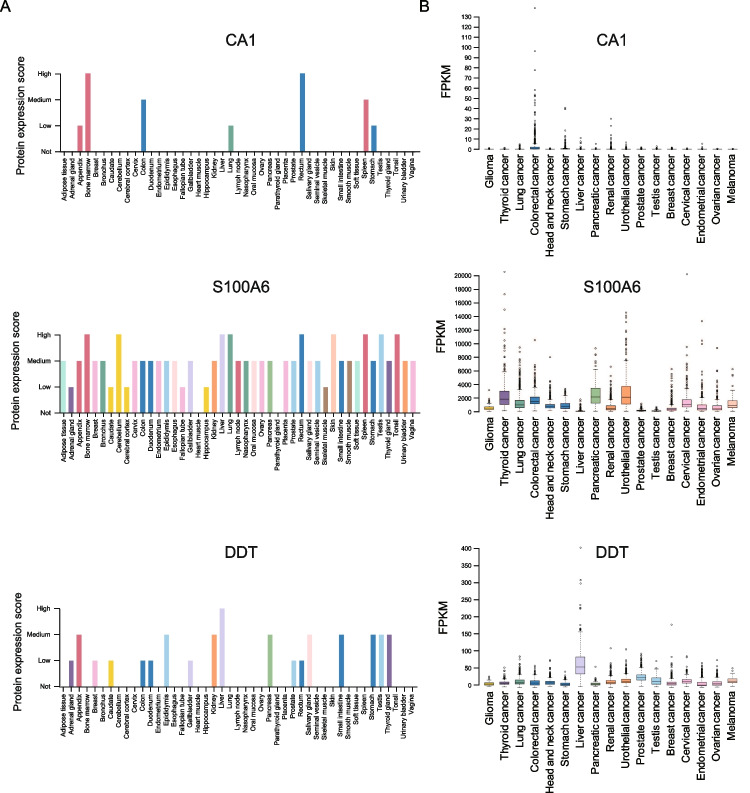
The protein and RNA expression of the marker across diverse tissues. **A** The protein expression score of CA1, S100A6, and DDT across diverse tissues based on HPA database. **B** The RNA expression of CA1, S100A6, and DDT across diverse tissues based on TCGA database

In addition, we also explored the enrichment of these proteins in different tissues and the association with different cancers. After matching with the Human Protein Atlas (HPA) database, we found CA1 (Carbonic Anhydrase 1), showed high expression in bone marrow and rectum tissues, and medium expression in colon and spleen tissues **(**Fig. 4A**)**. The S100A6 (S100 Calcium Binding Protein A6), was merely expressed in the 45 kinds of tissues, except in heart muscle and parathyroid gland, which showed high expression in many tissues (including bone marrow, cerebellum, liver, lung, rectum, skin, spleen, testis, and tonsil). As for the DDT (D-Dopachrome Tautomerase), it showed high expression in liver. Furthermore, according to the TCGA dataset, we found CA1 was enriched in colorectal cancer and stomach cancer; while S100A6 and DDT showed low cancer specificity, especially that S100A6 showed higher expression in urothelial cancer and DDT showed higher expression in liver cancer **(**Fig. 4B**)**. Our findings indicated that these markers could not only associated with T2DM, but also showed a certain association with cancer types.

## Discussion

Diabetes, as a complex disease, involves many alterations in many signaling pathways and biological processes during the development and progression [33–36]. Due to the multifactorial and multigenetic nature of the disease, the molecular biomarkers for T2DM are still needed to be identified and verified. With the development of the mass spectrometry (MS)-based proteomics, the application of MS in blood samples could further provide an optimal technology for biomarker identification [15, 25, 37–39]. Although many markers were identified and associated with T2DM [40–43], there was a low consistency of these biomarkers in different cohorts of T2DM, underscoring the importance of refining a set of reliable and stable predictive markers through the integration of diverse cohorts. Therefore, it is very necessary to integrate different cohorts to refine a group of reliable markers, which had a stable predictive effect.

In this study, we included two independent cohorts and incorporated the extracellular vesicle (EV) proteome data in the cohort 1 and the serum proteome data in the cohort 2. By analyzing these datasets, we identified the consistent biological alteration associated with T2DM in the two cohorts, of which, neutrophil degranulation, VEGF-VEGFR2 signaling pathway, metabolic pathways were activated in the T2DM; while immune system exhibited a decreased level in the T2DM. In addition, we also determined the potential hub proteins involved in the representative biological pathways of T2DM and HC. Finally, we identified the three proteins, including CA1, S100A6, and DDT, to distinguish T2DM patients from healthy controls. The predictive effect of the three proteins was further evaluated using AUC and accuracy metrics analysis, which was adopted in the biomarker identification and prediction model construction [44–46]. In this study, the predictive model, comprising the three proteins CA1, S100A6, and DDT, exhibited high prediction in both the training set and the testing set. These results indicated the predictive model's ability to accurately classify individuals with T2DM and healthy controls, suggesting a high level of both specificity and sensitivity. Merging two datasets from independent studies allowed for cross-validation of findings, reinforcing the reliability and reproducibility of identified markers, and potentially uncovering novel biomarkers that may have been overlooked in individual studies.

Additionally, we also explored the potential biological function and clinical potential of the three proteins (including CA1, S100A6, and DDT) as drug targets. CA1, as a member of the carbonic anhydrase family, plays a crucial role in catalyzing the reversible hydration of carbon dioxide, participating in metabolic process such as carbon disulfide oxidation respiration [47]. As reported, the carbonic anhydrases (CAs) were involved in the formation and progression of plaques in atherosclerosis, which was the main pathological change of the cardio-cerebrovascular disease companied by T2DM [48]. Researches showed that several drugs have been developed and approved for targeting and inhibiting CA1 in various clinical trials for different diseases. These drugs include Diclofenamide, which functions as an antiglaucomic agent [49]; Chlorothiazide, known for its role as an antihypertensive agent [50]; Zonisamide, used as an antiepileptic with anticonvulsant properties [51]; and Sulpiride, which functions as an antidepressant agent [52]. These drugs suggested the potential versatility of targeting CA1 for therapeutic interventions, offering promising avenues for addressing various diseases and conditions beyond T2DM. As for S100A6, a member of the S100 family of calcium-binding proteins [53], has been reported to be associated with T2DM development, contributing to hepato-pancreatic communication to reduce insulin production and promote the progression of T2DM in individuals with nonalcoholic fatty liver disease [54]. This might suggest the potential of S100A6 as a target for interventions aimed at modulating insulin dynamics and managing T2DM-related complications. As for DDT, the D-Dopachrome Tautomerase, was related to dopachrome isomerase activity and D-dopachrome decarboxylase activity [55]. Although some drugs for DDT were approved or investigated, such as carbon dioxide and androgen antagonists, the direct association between DDT protein and T2DM has not been reported in the previous literature. While the direct association of DDT with T2DM is novel, further validation of the robustness of these three proteins (CA1, S100A6, and DDT) as potential diagnostic biomarkers for T2DM is needed in larger sample sizes. Therefore, the association of the three proteins (CA1, S100A6, and DDT) in predicting T2DM should be verified in a relatively larger sample size to verify its robustness. The findings in this study revealed the three proteins could be used as the potential diagnostic biomarkers for T2DM.

In the field of T2DM management, a range of drugs such as Insulin, Metformin, Sulphonylureas, Meglitinides, Alpha-glucosidase inhibitors, Thiazolidinediones, DPP-4 inhibitors, Incretin mimetics, and Amylin analogues are employed to regulate blood sugar levels through various mechanisms [56–59]. Novel therapeutic strategies for T2DM are exploring strategies, such as direct insulin secretion stimulation, leveraging the incretin axis, hepatic glucose production suppression, and enhanced insulin sensitivity to tailor glycemic control for each patient while ensuring safety and preventing complications [60, 61]. The identification of promising drug targets like CA1, S100A6, and DDT further advances the precision medicine landscape in diabetes care, offering potential avenues for targeted therapeutic interventions and improved outcomes. Understanding the dual role of CA1, S100A6, and DDT as both diagnostic biomarkers and potential therapeutic targets in T2DM is pivotal. Exploring the functional roles of these proteins in disease progression could pave the way for innovative treatment modalities that target T2DM's specific molecular underpinnings. Future research should focus on investigating the therapeutic significance of these proteins and their manipulation in drug development to enhance diabetes management.

In conclusion, we identified the potential biological pathways associated with T2DM, and found the consistency in the two cohorts, which further enhanced the stability of the markers for T2DM. Despite the small number of patients, the study’s strength lies in the fact our study is the combined analysis of two cohorts to refine a group of potential markers for T2DM. Additionally, the potential markers revealed in this study should be further verified by the other methods besides MS-proteomics in blood samples. Also, the link between clinical features and the proteomics data, should be clarified for the implementation of clinical practice.

## Conclusion

Our study represents a significant advancement in the field of Type-2 diabetes mellitus (T2DM) research by employing integrative proteomic analysis to refine a panel of potential protein markers for T2DM. This innovative approach enhances the understanding of the molecular landscape underlying T2DM and offers novel insights into the disease pathology. By integrating data from two distinct cohorts and utilizing advanced proteomic techniques, we have identified a specific group of proteins, including CA1, S100A6, and DDT, that demonstrate consistent upregulation in T2DM patients across both cohorts.

The novelty of our study is the comprehensive integration of proteomic data to refine and validate a set of protein markers with high predictive accuracy for T2DM. These refined markers hold promise for improving diagnostic precision and may serve as valuable targets for clinical translation and personalized treatment strategies in the management of T2DM. By focusing on the refinement and validation of these specific protein markers, our research contributes to develop more reliable and effective diagnostic tools for T2DM.

The refined protein panel identified in our study offers a promising avenue for further research and potential clinical applications. By enhancing the accuracy and reliability of T2DM markers, our findings pave the way for more targeted and personalized approaches to T2DM diagnosis and management. This work underscores the importance of integrative proteomic analysis in refining biomarkers for complex diseases like T2DM and highlights the potential for improved clinical outcomes and patient care through the identification of reliable protein markers.

## Supplementary Information

Below is the link to the electronic supplementary material.

Supplementary file1 (XLSX 31 KB)

Supplementary file2 (XLSX 15 KB)

## Data Availability

The ConsensusPathDB (CPDB) molecular interaction data could be accessed at http://www.consensuspathdb.org/. The protein and RNA expression data from Human Protein Atlas (HPA) could be accessed at https://www.proteinatlas.org/. The remaining data are available within the Article and Supplementary Information. The data that support the findings of this study are available on reasonable request from the corresponding author.
